# A systematic survey of the response of a model NF-κB signalling pathway to TNFα stimulation

**DOI:** 10.1016/j.jtbi.2011.12.014

**Published:** 2012-03-21

**Authors:** Yunjiao Wang, Pawel Paszek, Caroline A. Horton, Hong Yue, Michael R.H. White, Douglas B. Kell, Mark R. Muldoon, David S. Broomhead

**Affiliations:** a Manchester Interdisciplinary Biocentre, University of Manchester, Manchester M1 7DN, UK; b Faculty of Life Sciences, University of Manchester, M13 9PL, UK; c School of Mathematics, University of Manchester, M13 9PL, UK; d Center for Cell Imaging, School of Biological Sciences, Bioscience Research Building, Crown Street, Liverpool L69 7ZB, UK; e Department of Electronic and Electrical Engineering, University of Strathclyde, Graham Hills Building, Glasgow G1 1XW, Scotland, UK; f School of Chemistry, University of Manchester, Manchester M1 7DN, UK; g Mathematical Bioscience Institute, Ohio State University, Columbus, OH 43210, USA

**Keywords:** NF-κB signalling pathway, Parameter sensitivity, Bifurcation analysis, Oscillations

## Abstract

White's lab established that strong, continuous stimulation with tumour necrosis factor-α (TNFα) can induce sustained oscillations in the subcellular localisation of the transcription factor nuclear factor κB (NF-κB). But the intensity of the TNFα signal varies substantially, from picomolar in the blood plasma of healthy organisms to nanomolar in diseased states. We report on a systematic survey using computational bifurcation theory to explore the relationship between the intensity of TNFα stimulation and the existence of sustained NF-κB oscillations. Using a deterministic model developed by Ashall et al. in 2009, we find that the system's responses to TNFα are characterised by a supercritical Hopf bifurcation point: above a critical intensity of TNFα the system exhibits sustained oscillations in NF-kB localisation. For TNFα below this critical value, damped oscillations are observed. This picture depends, however, on the values of the model's other parameters. When the values of certain reaction rates are altered the response of the signalling pathway to TNFα stimulation changes: in addition to the sustained oscillations induced by high-dose stimulation, a second oscillatory regime appears at much lower doses. Finally, we define scores to quantify the sensitivity of the dynamics of the system to variation in its parameters and use these scores to establish that the qualitative dynamics are most sensitive to the details of NF-κB mediated gene transcription.

## Highlights

► The system's responses to TNFa are characterised by a supercritical Hopf bifurcation. ► A second oscillatory regime appears at much lower doses at certain reaction rates. ► The qualitative dynamics are most sensitive to the details of NF-kB mediated gene transcription.

## 1 Introduction

The transcription factor NF-κB is critical to the control of response to cellular stress and is involved in the regulation of cell-cycle/growth, apoptosis, inflammation and immunity (Hayden and Ghosh, 2008; Ghosh et al., 1998; Pahl, 1999; Gerondakis et al., 2006; Hoffmann and Baltimore, 2006; Hayden et al., 2006; Gerondakis et al., 2006; Bassères and Baldwin, 2006; Dutta et al., 2006). NF-κB is composed of homo- or hetero-dimers, with the ubiquitously expressed RelA:p50 hetero-dimer being the primary inflammatory mediator (Hoffmann and Baltimore, 2006).

In the absence of any stimulus, NF-κB is held in an inactive state in the cytoplasm where is sequestered by association with inhibitory κB (IκB) proteins including IκBα, IκBβ, and IκBϵ. In response to stimulation by cytokines, including TNFα, activated IκB kinases (IKKs) phosphorylate the IκB proteins, targeting them for degradation via the ubiquitin-proteasome pathway (Ghosh et al., 1998). Liberated NF-κB translocates to the nucleus and regulates target gene transcription, including highly inducible IκBα and zinc finger protein A20 genes (Scott et al., 1993; Song et al., 1996). This transcriptional control constitutes negative feedback regulation (Lipniacki et al., 2004). Newly synthesised IκB binds to nuclear NF-κB, leading to export of the complex to the cytoplasm (Arenzana-Seisdedos et al., 1995), while A20 inhibits the NF-κB signalling cascade (Lipniacki et al., 2004; Wertz et al., 2004), acting upstream of IKK.

Coupled negative feedback loops may lead to oscillations, and, indeed, Hoffmann et al. (2002) used electromobility shift assay (EMSA) to observe damped oscillations in NF-κB nuclear activity at the population level. Subsequently White's lab (Nelson et al., 2004) observed sustained oscillations in NF-κB nuclear localisation at the single-cell level using fluorescence microscopy. In these experiments cells were stimulated continuously with a high dose of TNFα (10 ng/ml). In contrast, blood plasma measurements suggest that physiological concentrations of TNFα may be considerably lower (Nakai et al., 1999; Damas et al., 1989; Matalka et al., 2005). Recent single-cell data from White's and other labs demonstrated that a fraction of cells in the population can respond to concentrations of TNFα as low as few pg/ml, albeit with an apparently stochastic delay (Turner et al., 2010; Tay et al., 2010; Kingeter et al., 2010).

Dynamical responses of the NF-κB system regulate many physiological processes associated with inflammatory signalling. Frequency of oscillations, their persistence and other characteristics are fundamental in controlling patterns of downstream gene expression (Ashall et al., 2009; Tay et al., 2010). Therefore, a quantitative understanding of systems responses at physiological doses of the stimulus is required to elucidate the biological function of these dynamics. Here we use numerical bifurcation theory to survey systematically the dynamical responses of a model of the NF-κB system developed by Ashall et al. (2009). First, in Section 2 we consider the one parameter bifurcation problem associated with variation in the intensity of TNFα stimulation. Of course, the dynamics of the system would also be expected to change if one perturbed, for example, the reaction rates. In order to understand the influence of the variation of these rates on the qualitative dynamics of the system, we also carried out an extensive programme of two-parameter bifurcation studies that are discussed in Section 3. Finally, in Section 4, we define a parameter sensitivity score to quantify the sensitivity of the dynamics to variations in each parameter and use these scores to inform a discussion of further model development.

## 2 One-parameter bifurcations

**Fig. 1:**
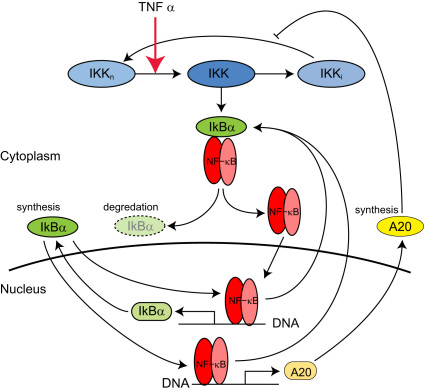
A graphical representation of the model NF-κB signalling network detailed in Appendix A. Here ovals represent proteins and the two rounded rectangles represent messenger RNAs whose production is regulated by NF-κB.

The deterministic model in Ashall et al. (2009) is among the more detailed models of the NF-κB signalling system and includes, for example, explicit terms for mRNA concentration. It stands in contrast to reduced models such as those of Krishna et al. (2006) and Fonslet et al. (2007) that aim to capture essential mechanisms at the expense of some biochemical detail. It consists of two negative feedback loops: one in which NF-κB transcriptionally regulates its inhibitor IκBα and another in which it regulates expression of A20 protein, which acts indirectly to suppress the kinase IKK involved in liberating NF-κB from IκBα–NF-κB complexes. The model network is illustrated in Fig. 1 and specified in detail by a system of ordinary differential equations (ODEs) listed in Appendix A along with the associated parameters, which are listed in Table 1.

This model assumes that IKK exists in one of three forms: neutral denoted by IKK_*n*_ (the form that can be activated), active denoted by IKK (the form that phosphorylates IκBα and its complexes) and inactive denoted by IKK_*i*_ (a state that cannot be activated). The model also assumes that inactive IKK can be recycled into active IKK and that A20 acts by inhibiting this recycling process.

The full model contains 14 ordinary differential equations, including equations governing the concentrations of the three forms of IKK; the free forms of IκBα, NF-κB and A20; the IκBα–NF-κB complex and the phosphorylated forms of IκBα and IκBα–NF-κB; and A20 and IκBα transcript. Most biochemical reactions are modelled with mass-action kinetics: the exceptions are the NF-κB-mediated transcriptional regulation of the A20 and IκB genes and the action of A20 on IKK recycling. The regulation of gene expression by NF-κB is modelled by an increasing Hill function of the form

(1) $$f(x)=\frac{\beta x^{h}}{k^{h}+x^{h}}$$

where *x* represents the concentration of the transcription factor, *k* is the concentration required to produce a half-maximal response, and β gives the maximal rate of transcription. The parameter *h* is often called the *Hill coefficient* and it regulates the nonlinearity of the response. The graph of *f*(*x*) in (1) is an increasing S-shaped curve that becomes more step-like as one increases *h*. The inhibition of IKK_*i*_ to IKK_*i*_ cycling mediated by A20 is modelled by a decreasing Hill function

(2) $$\frac{\beta }{k+x}$$

Although the model describes the temporal evolution of 14 chemical concentrations, there are really only 12 independent quantities as the system has two conservation laws. First, NF-κB is neither created nor destroyed, so the total amount of NF-κB (comprising free NF-κB and its complexes, both cytoplasmic and nuclear) remains constant. Similarly, total IKK (encompassing the neutral, active and inactive forms) is also conserved. Additionally, the concentration of phosphorylated IκBα (pIkB α in the notation of Ashall et al., 2009) does not appear in any of the ODEs except the one involving its own degradation. Thus we can ignore it and, taking account of the two conservation laws, reduce the model to a system of 11 ODEs: these are given in Appendix A.

The bifurcation and sensitivity analyses reported below are based on this reduced system, in which the intensity of TNFα stimulation, denoted by *T*_*R*_, and the total amounts of NF-κB and IKK are treated as parameters. The latter two are denoted by totalNFkB and totalIKK, respectively. The reduced model – when used with the parameters in Table 1 – captures the two main features observed in the single cell experimental data in Ashall et al. (2009) and Nelson et al. (2004): (i) continuous, high dose (*T*_*R*_=1) TNFα stimulation leads to sustained oscillations with a period near 100 min and (ii) in the absence of stimulation, the system remains in a steady state and does not oscillate.

### 2.1 Varying the dose

In the model, TNFα activates NF-κB via activation of IKK. Eqs. (3) describe the rates of the neutral and active IKK

$${\mathrm{IKK}}_{n}(t)\prime =k_{p}\cdot (\text{totalIKK}-{\mathrm{IKK}}_{n}(t)-\mathrm{IKK}(t))\cdot \frac{k_{\mathrm{bA}20}}{k_{\mathrm{bA}20}+A20(t)\cdot T_{R}}-T_{R}\cdot k_{a}\cdot \mathrm{IKK}(t)$$

(3) $$\mathrm{IKK}(t)\prime =T_{R}\cdot k_{a}\cdot {\mathrm{IKK}}_{n}(t)-k_{i}\cdot \mathrm{IKK}(t)$$

where $k_{⁎}$ are parameters, A20(t) is the concentration of the protein A20 and, in Ashall et al. (2009), *T*_*R*_ indicates the presence or absence of high dose (10 ng/ml) TNFα stimulation: *T*_*R*_=1 when TNFα is present and *T*_*R*_=0 when it is absent. It is thus natural to generalise the role of *T*_*R*_ and to use values $0\leq T_{R}\leq 1$ to represent the range of intensities of TNFα stimulation. Note that we do not assume that the value of *T*_*R*_ depends linearly on the concentration of TNFα, but only that the relationship is monotone and saturates at *T*_*R*_=1 for high dose stimulation.

Now consider *T*_*R*_ as a bifurcation parameter. The parameter totalNFkB corresponds to the initial concentration of IκBα–NFκB complex and is set to 0.08μM, while the concentrations of all other forms of NF-κB are set to zero. Similarly, totalIKK is set by assigning the initial concentration of neutral IKK to ${\mathrm{IKK}}_{n}=0.08\;\mu M$ and the concentrations of all other forms of IKK to zero. The remaining parameters are held at the values used in Ashall et al. (2009).

**Fig. 2:**
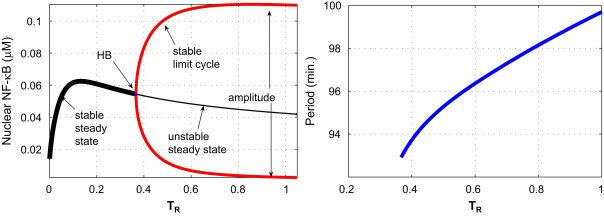
Left panel: bifurcation diagram for *T*_*R*_, where the thick black curve represents a branch of steady states, the thin black curve represents a branch of unstable steady states and the red curve represents a branch of limit cycles. The upper part of the red curve shows the peak values of the oscillations while the lower part shows the troughs. Right panel: period of the limit cycle as a function of *T*_*R*_.

Starting from the steady state equilibrium associated with unstimulated cells (*T*_*R*_=0), the numerical bifurcation using XPPAUT indicates a branch of steady state solutions that changes stability by way of a supercritical Hopf bifurcation (HB) when $T_{R}\equiv T_{R}^{⋆}\approx 0.366$. This means that, with other parameters held fixed, if $T_{R}<0.366$ any initial configuration of concentrations will undergo damped oscillations and eventually settle into a steady state with constant concentrations. But if $T_{R}>0.366$, the system will evolve into sustained oscillations. This is illustrated in the left panel of Fig. 2, while the right panel shows the *T*_*R*_ dependence of the period of oscillation.

Sustained oscillations eventually disappear via a second Hopf bifurcation at *T*_*R*_=156, but such a large value of *T*_*R*_ is not biologically meaningful. We emphasise again that *T*_*R*_ is not proportional to TNFα concentration: rather, it indicates the extent to which the signalling machinery is saturated. Single-cell measurements show that the system is already saturated at doses of 10 ng/ml (Tay et al., 2010; Turner et al., 2010) so we have restricted our analysis to values of *T*_*R*_ between 0 and 1.

From the right panel of Fig. 2 we see that the period of the limit cycle varies over a small range, increasing from 93 min to 99 min as *T*_*R*_ increases from 0.366 to 1. We note that when $T_{R}<0.366$, experimental observations might still appear to indicate the presence of oscillations, as all the stable steady states in the branch are foci (the Jacobian along the branch of steady state has complex eigenvalues). For levels of stimulation in this particular region, the oscillations will damp away exponentially, but – especially for *T*_*R*_ near the HB-point – the damping rate is very small, so that oscillations in this regime, though decaying slowly, may appear to persist throughout the period of observation. Additionally, molecular noise can support sustained stochastic oscillations even for *T*_*R*_ below the HB point: see Turner et al. (2010).

## 3 Two-parameter bifurcations

As the values of parameters in the model are determined either by direct measurement or via model-fitting, both of which are subject to error, it is natural to ask how the structure of the one-parameter bifurcation is influenced by the changes to the other parameters. One can begin to address this question by performing two-parameter bifurcation analyses. Consider, for example, the parameter totalIKK: in Section 2.1 we obtained a one-parameter bifurcation diagram for the case totalIKK=0.08μM. Now if we change value of totalIKK by a small amount and recompute the one-parameter bifurcation diagram, then typically we will get a new diagram similar to Fig. 2, but with the HB-point in a slightly different position. This suggests that the one-parameter bifurcation diagram could be summarised in terms of the existence and location of the HB-point. Further, the dependence of the HB-point's position on totalIKK can be summarised by a two-parameter bifurcation diagram, which is a projection of all bifurcation points into the (*T*_*R*_, totalIKK)-plane. Such a diagram shows how the bifurcation point moves with variation in a second parameter value, which here is the value of totalIKK.

Two-parameter bifurcation diagrams can also be computed with the numerical bifurcation package AUTO (Doedel et al., 2000): Fig. 3(left) is the two-parameter diagram for *T*_*R*_ and totalIKK. In the left panel we can see that near the original HB-point, a cell with a higher level of totalIKK would require stronger TNFα stimulation to support sustained oscillations. But the slope reverses for totalIKK<0.014μM: there a cell with a *lower* concentration of total IKK needs stronger intensity of TNFα stimulation to support oscillations. And if totalIKK<0.002μM, then no sustained oscillations occur for any level of TNFα stimulation.

**Fig. 3:**
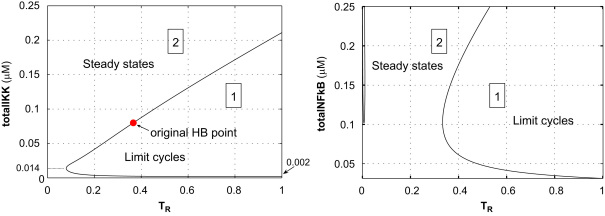
The two-parameter bifurcation diagrams for *T*_*R*_ and totalIKK (left panel) and *T*_*R*_ and totalNFkB (right panel). Here each point on the curves represents a HB-point and the curves divide the parameter spaces into two types of region: there is a limit cycle for each pair of the parameters in Region 1, in which and there is a non-oscillatory steady-state for each pair of parameters in Region 2.

**Fig. 4:**
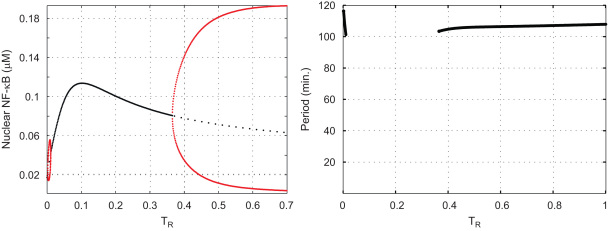
The bifurcation diagram for *T*_*R*_ when totalNFkB=0.15μM: there are three HB-points at $T_{R}=0.002$, 0.01 and 0.366, respectively. Sustained oscillations occur for values of *T*_*R*_ in either of the intervals (0.002, 0.01) and (0.366, 1]. The right panel shows the period of these oscillations.

One might expect that if the perturbation to the second parameter is large, novel dynamical behaviour may appear. This happens when we increase the value of total NF-κB from 0.08μM to a value greater than 0.12μM: see the right panel in Fig. 3. When, for example, totalNFkB=0.15μM, the corresponding one-parameter bifurcation diagram, which appears in the left panel of Fig. 4, has three HB-points.

The system has sustained oscillations when *T*_*R*_ lies in either of the intervals (0.002,0.01) or (0.366,1]. The amplitude of the oscillations is quite small when *T*_*R*_ is in the interval (0.002,0.01) with a period in the range of (101,114)min.

Bifurcation structures similar to those in Fig. 4 – with three Hopf bifurcations – also appear in the two-parameter diagrams for *T*_*R*_ and each of the parameters *h*, totalNFkB, *k*, *k*_*itria*_, *k*_*tria*_, *k*_*i*1_, $k_{a_{1}}$, *k*_*degf*_, *k*_*c*3_ and *k*_*d*1_. All remaining two-parameter diagrams appear in Appendix C, while Table 1 lists the ranges of parameter values over which the one-parameter bifurcation diagram in *T*_*R*_ retains the same structure as is illustrated in Fig. 2—that is, a single supercritical Hopf bifurcation and no others.

**Fig. B1:**
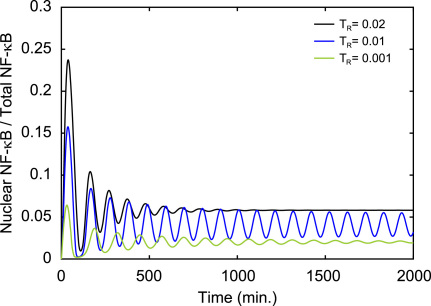
A typical experiment tracks single cells for ≈600 min, which is long enough to distinguish the low-dose oscillatory regime (blue curve) from the exponentially damped oscillations that occur at slightly higher (black curve) and lower (green curve) levels of stimulation. The model here is the same as that depicted in Fig. 4 and has totalNFkB=0.15μM. (For interpretation of the references to color in this figure legend, the reader is referred to the web version of this article.)

The bifurcation diagrams in Appendix C make it clear that a wide range of parametric variation produces only two types of two-parameter bifurcation diagrams. One type topologically resembles the diagram on the left panel of Fig. 3 where, for a given value of the second parameter, there is a unique critical value of *T*_*R*_ separating the levels for stimulation that induce sustained oscillations for those that produce only damped oscillation. The second type of bifurcation diagram resembles that in Fig. 3: variation in of second parameter can lead to oscillating behaviour associated with the high levels of TNFα stimulation, but for suitable parameter values a second, separate oscillatory regime exists for very low-intensity TNFα stimulation. These low-dose oscillations are isolated from the high-dose oscillations in the sense that there are intermediate values of *T*_*R*_ for which no oscillations occur. Further, the low-dose oscillations are readily distinguishable from those in the adjacent damped regime: see Fig. B1 in Appendix B.

## 4 Parameter sensitivity analysis

In this section we define a *sensitivity score* to quantify the sensitivity of the model to changes in the parameters. In the previous section we observed that for parameters in the ranges listed in Table 1, the one-parameter bifurcation diagram in *T*_*R*_ retains the qualitative structure illustrated in Fig. 2: there is a single HB-point. Our sensitivity score measures the impact of variation in the other parameters on the location of the HB-point in the associated one-dimensional *T*_*R*_ diagram. In addition, as changes in the period of the oscillation may lead to changes in the pattern gene expression (Ashall et al., 2009), we also investigate the sensitivity of the period to variation of the parameters.

Consider a parameter (other than *T*_*R*_) whose unperturbed value is $\mu_{0}$. The associated sensitivity score is defined as the average value of $\left(\partial T_{R}/T_{R}^{⁎})/(\partial \mu /\mu^{0}\right)$ with μ in a region

$$\mu_{0}(1-\theta )\leq \mu \leq \mu_{0}(1+\theta ),$$

where θ<1 is a prescribed fractional change in the parameter and $T_{R}^{⋆}$ is the value of *T*_*R*_ at the Hopf bifurcation point as described in Section 2.1.

We take the following steps to compute the score:

1. Read the pairs of $\left(T_{R},\mu \right)$ from the two-parameter bifurcation diagram over the region $\mu_{0}(1-\theta )\leq \mu \leq \mu_{0}(1+\theta )$: this bifurcation curve and the corresponding $\left(T_{R},\;\mu \right)$ pairs comes from AUTO.
2. Use piecewise cubic Hermite interpolation to fit a continuous function to the $\left(T_{R},\;\mu \right)$ pairs.
3. Sample m=2l+1 uniformly-spaced points from the interpolated curve, where the number of sampling points *m* is the same for all parameters. Denote these points as: $P_{-l},P_{-l+1},\ldots ,P_{-1},P_{0},P_{1},\ldots ,P_{l}$, with $P_{j}=(\mu^{j},\;T_{R}^{j})$ for −l≤j≤l.
4. The sensitivity score ${\mathrm{SS}}_{\mu }$ is then defined by the approximate integral
  (4) $${\mathrm{SS}}_{\mu }=\frac{1}{2\;l}\overset{l-1}{\underset{i=-l}{\sum }}\left(\frac{T_{R}^{i+1}-T_{R}^{i}}{\mu^{i+1}-\mu^{i}}\right)\left(\frac{\mu_{0}}{T_{R}^{⋆}}\right)$$
  where $\mu_{0}$ is the parameter value used in Ashall et al. (2009) and $T_{R}^{⋆}$ is the critical value from Fig. 2.

**Fig. 5:**
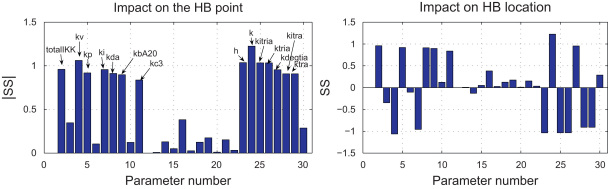
Sensitivity scores for the 26 reaction rates and the two concentration parameters (totalNFkB and totalIKK) constructed using Eq. (4) with θ=0.1: (left) absolute value of the sensitivity score against parameter number (see Table 1) and (right) sensitivity score against the index of parameters.

We computed sensitivity scores by varying each parameter individually by a fraction of θ=0.1: they are displayed in Fig. 5, where the numbering of the parameters is the same as that used in Table 1. We found that the parts of the two-parameter bifurcation curves spanned by these 10% variations were well-approximated by line segments, so based our scores on samples of *m*=21 points for each parameter, which is more than sufficient to guarantee convergence of the integrals.

The scores are clearly divided into two groups: those with higher scores and those with lower ones. Using a threshold value of |SS|≥0.5, parameters with the higher sensitivity scores are listed in Table 2, which shows that the system is most sensitive to variation of the parameters related to transcription and translation of IκBα and A20 and to those influencing IKK activity. These results are consistent with those obtained earlier by Yue et al. (2006, 2007, 2008) and Ihekwaba et al. (2004). The analysis in these earlier papers was based on a deterministic model developed by Hoffmann et al. (2002), which includes only the core NF-κB and IκB feedback loops. While Yue and Ihekwaba analysed the sensitivity of a certain transient response to the variations in the model's parameters, we have here used both a more recent model and have studied the sensitivity of long-time dynamical behaviour—the question of whether there are stable oscillations or not. Our results thus support and complement the earlier work, bolstering their finding about the high sensitivity of the dynamics to variation in those parameters related to transcription and translation of IκBα as well as those influencing IKK activity. Further, our analysis indicates a role for the parameters associated with the transcription and translation of A20 that is comparable in importance to those influencing production and dynamics of IκBα.

Since the sensitivity score defined here has the same sign as the mean values of the slopes along the two-parameter bifurcation curves, and as the relevant segments of the curves are nearly linear, the sign of the scores provides further information. For example, note that *k*_*tria*_, *k*_*tra*_, *k*_*itria*_ and *k*_*itra*_ all have negative scores. This implies that the faster the transcription or translation of IκBα and A20, the lower the dose of TNFα needed to induce sustained oscillations. Similar properties can be read off for other parameters from Fig. 5.

**Fig. 6:**
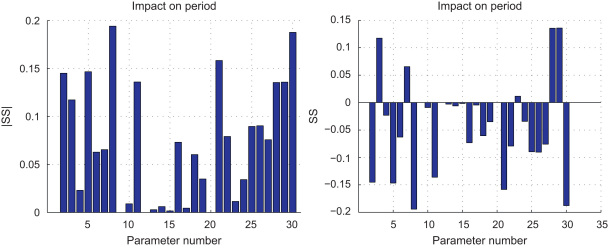
Scores measuring the sensitivity of the period of the NF-κB oscillations to the changes in parameters: (left) absolute value of sensitivity and (right) sensitivity score against parameter index.

As pointed out in Ashall et al. (2009), the period of the oscillations plays an important role in selecting which of the genes that NF-κB targets are expressed. We thus also considered the sensitivity of the period of the NF-κB oscillations (while *T*_*R*_=1) to the changes in parameters. That is, fixing *T*_*R*_=1 and varying each of the other parameters by 10%, we compute the sensitivity scores by substituting *T*_*R*_ by the value of the period at the corresponding parameters in (4). Using the same sampling principles, we obtain the resulting scores that are shown in Fig. 6.

Note that all scores in Fig. 6 are much smaller than 0.5, which is the threshold we used for distinguishing the higher and lower score groups in Fig. 5. This that, compared to the location of the Hopf bifurcation, the oscillatory period is much less sensitive to the parameters.

## 5 Discussion

The transcription factor NF-κB is pivotal in controlling body's innate immunity, but can also contribute to carcinogenesis (Karin et al., 2002). Live-cell imaging has established that NF-κB undergoes sustained oscillations in cellular localisation that can be induced by continuous, high doses of TNFα stimulation (Ashall et al., 2009; Nelson et al., 2004). More recently, the remarkable ability of the NF-κB system to oscillate in response to much lower, physiological doses of TNFα has also been demonstrated experimentally (Turner et al., 2010; Tay et al., 2010; Kingeter et al., 2010). The data from White's lab (Turner et al., 2010) showed that individual SK-N-AS neuroblastoma cells may oscillate in response to stimulation with doses as low as picomolar TNFα, though this response is apparently probabilistic, with the fraction of responsive cells declining with dose.

Other studies using different cell types showed similar stochastic activation of the pathway. However, oscillations of the transcription factor at lower doses were less apparent (Tay et al., 2010; Kingeter et al., 2010). Stochastic models of the system suggest that probabilistic nature of low-dose responses may arise from the noisy activation of the transduction pathway leading from the TNFα receptor to IKK module (Turner et al., 2010) and more specifically, to the limited availability of TNFα trimers for receptor binding (Lipniacki et al., 2007; Tay et al., 2010). Because of their stochastic nature, none of these models is straightforwardly amenable to the sort of systematic bifurcation analyses reported above.

Here, we have surveyed the qualitative behaviour of a recent deterministic model of the NF-κB system (Ashall et al., 2009), and used bifurcation analysis to characterise all possible responses to TNFα stimulation with the intensity in the range of 0–10 ng/ml. Further, we have quantified the impact of variation in the remaining parameters on the level of stimulation required to induce oscillations. Using the parameter *T*_*R*_ – a proxy for the intensity of TNFα stimulation – as bifurcation parameter, we found that sustained oscillations appear via a supercritical Hopf bifurcation. That is, there exists a critical intensity of stimulation $T_{R}^{⋆}$ such that sustained oscillations will occur whenever $T_{R}>T_{R}^{⋆}$, while only damped oscillations will occur for $T_{R}<T_{R}^{⋆}$. Further, a similar bifurcation structure persists across a wide range of variation in most of the model's other parameters. There is, however, a group of parameters – about a third of those appearing in the model – for which sufficiently large modulations introduce the possibility of a second, distinct sort of periodic oscillation appearing at very low levels of stimulation.

Building on our bifurcation analyses, we performed sensitivity analyses to quantify the effect of parameter variation on the location of the supercritical Hopf bifurcation and on the period of the oscillation. The location of the bifurcation proved highly sensitive to variation in those parameters associated with transcription and translation of IκBα and A20 as well as those associated with IKK activity, a result consistent with previous work (Ihekwaba et al., 2004; Yue et al., 2006, 2007, 2008). By contrast, the period was much less sensitive to parametric variation.

The low-dose limit-cycle regime described above appears for $T_{R}<0.01$, which corresponds to levels of stimulation that induce less than 1% of a saturating response. An oscillatory response to such feeble stimulation offers a tantalising suggestion of a connection to data from low-dose experiments, but there are a number of obstacles to making this connection concrete. As we emphasised in Section 2, our main bifurcation parameter *T*_*R*_ is only a proxy for the intensity of TNFα stimulation: it is an abstraction representing the consequences of that part of the signalling cascade that extends from the receptors at the cell's surface down to IKK. A modest extension to the model might treat the upstream part of the signal transduction chain as a Hill function (1) whose input would be extracellular TNFα and whose output would play the role of *T*_*R*_. This suggests an explanation for the tenfold range of *T*_*R*_ variation over which the current model predicts no oscillations at all: these values could correspond to the step in the Hill function and thus to a narrow range of TNFα concentrations that might prove difficult to locate experimentally. But as neither we nor our collaborators have data that would allow us to fit such an extended model, we have not pursued it any further.

**Fig. C1:**
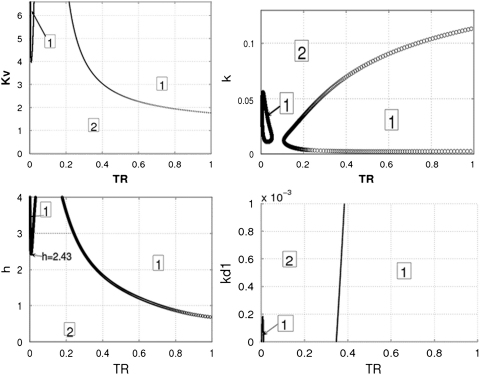
The two-parameter bifurcation diagrams for *T*_*R*_ and *k*_*v*_, *k*_*d*1_, *h* and *k*, in which the curves trace out the locations of Hopf bifurcation points. These curves divide the parameter space into two types of regions: limit cycle region is marked by 1 and steady-state region marked by 2.

**Fig. C2:**
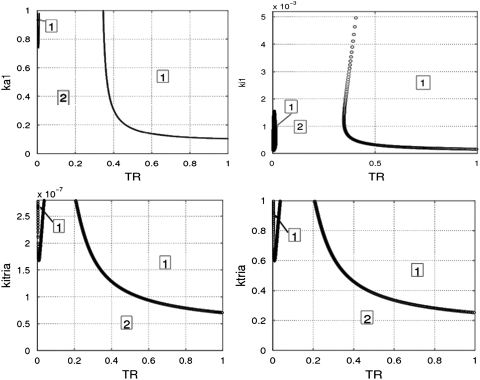
The two-parameter bifurcation diagrams for *T*_*R*_ and *k*_*a*1_, *k*_*i*1_, *k*_*itria*_ and *k*_*tria*_, in which the curves trace out the locations of Hopf bifurcation points. These curves divide the parameter space into two types of regions: limit cycle region is marked by 1 and steady-state region marked by 2.

The emergence of the low-dose oscillatory regime also depends on modulation of some of the model's other parameters, including expression levels of NF-κB and IKK, rates of transcription and transport among others (Figs. 3, C1 and C2). Data available in the literature show that during carcinogenesis many components of the NF-κB signalling systems are changed or mutated in such a way that the activity of the transcription factor is increased (for example in multiple myeloma, Annunziata et al., 2007; lung cancer, Tang et al., 2006; and colon cancer, Charalambous et al., 2009). These measurements are not really quantitative, and thus direct comparison with our model is not possible, but one could perhaps speculate whether during cancer progression parameters of the NF-κB system change in such a way as to promote anomalous limit cycle oscillations in a low-dose tissue micro-environment. This could enable more efficient proliferation of cells, perhaps by driving oscillation-dependent interactions with a cell cycle system – for example, via cyclin D (Sée et al., 2004) – that are inhibited or not activated in healthy tissue.

## 6 Methods

Bifurcation diagrams were prepared with XPPAUT (Ermentrout, 2002) and the command line interface Rauto (Schilder, 2007): both rest on the powerful numerical bifurcation package AUTO (Doedel et al., 2000). Sensitivity scores were computed with MATLAB R2006b: code is available on request.

## Figures and Tables

**Fig. C3:**
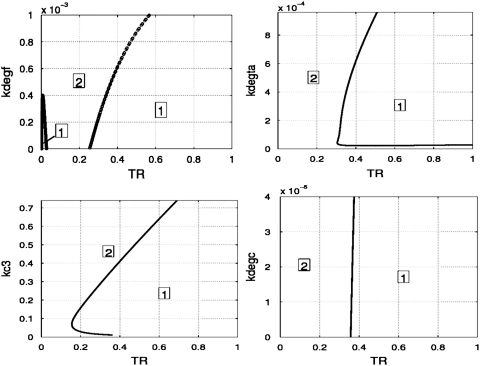
The two-parameter bifurcation diagrams for *T*_*R*_ and *k*_*degf*_, *k*_*degta*_, *k*_*c*3_ and *k*_*degc*_, in which the curves trace out the locations of Hopf bifurcation points. These curves divide the parameter space into two types of regions: limit cycle region is marked by 1 and steady-state region marked by 2.

**Fig. C4:**
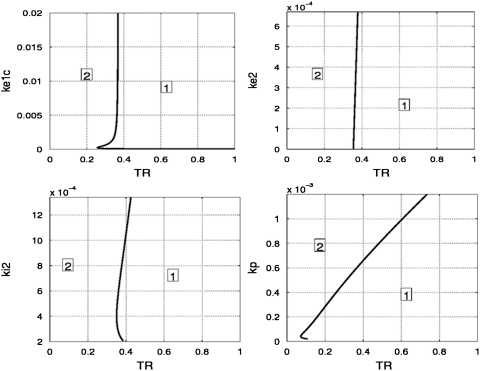
The two-parameter bifurcation diagrams for *T*_*R*_ and $k_{e1c}$, *k*_*e*2_, *k*_*i*2_ and *k*_*p*_, in which the curves trace out the locations of Hopf bifurcation points. These curves divide the parameter space into two types of regions: limit cycle region is marked by 1 and steady-state region marked by 2.

**Fig. C5:**
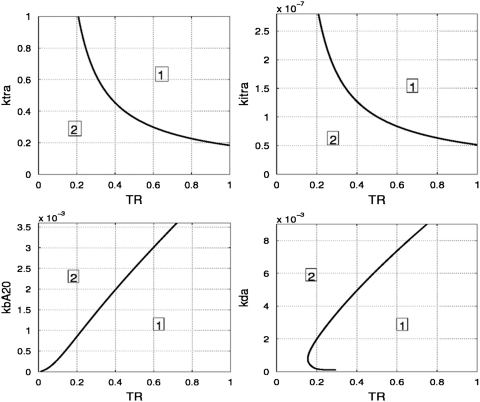
The two-parameter bifurcation diagrams for *T*_*R*_ and *k*_*tra*_, *k*_*itra*_, $k_{\mathrm{bA}20}$ and *k*_*da*_, in which the curves trace out the locations of Hopf bifurcation points. These curves divide the parameter space into two types of regions: limit cycle region is marked by 1 and steady-state region marked by 2.

**Fig. C6:**
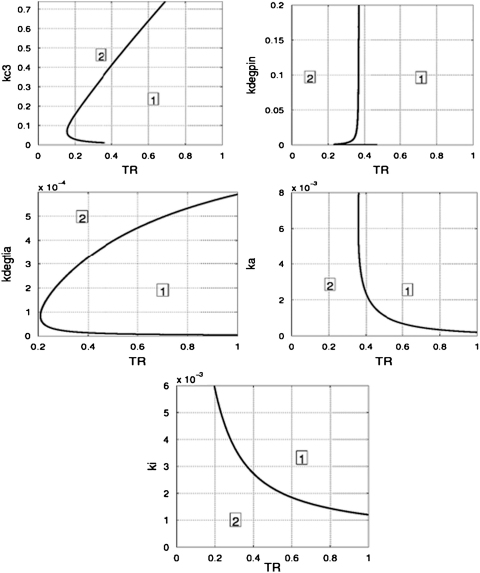
The two-parameter bifurcation diagrams for *T*_*R*_ and *k*_*c*3_, *k*_*degpin*_, *k*_*degtia*_, *k*_*a*_ and *k*_*i*_, in which the curves trace out the locations of Hopf bifurcation points. These curves divide the parameter space into two types of regions: limit cycle region is marked by 1 and steady-state region marked by 2.

**Table 1:** Ranges of model parameters and percentages-of-range for which the one-parameter bifurcation diagram in *T*_*R*_ has the same topology as that in Fig. 2.

Parameter		Range surveyed	Value in model	% one HB-point
Num.	Name			
1	*T*_*R*_	(0, 1)	1.0	—
2	totIKK	(0.001943, 0.21102)	0.08	97.6
3	totNFkB	(0.032147, 0.1 )	0.08	59.8
4	*k*_*v*_	(1.7658, 5.7363)	3.3	46.5
5	*k*_*p*_	(1.00E−05, 0.0015968)	0.0006	98.3
6	*k*_*a*_	(0.00010279, 2.9586)	0.004	97.4

7	*k*_*i*_	(0.0007336, 0.11817)	0.003	75.5
8	*k*_*da*_	(9.48E−05, 0.021019)	0.0045	97.9
9	$k_{\mathrm{bA}20}$	(6.81E−06, 0.0093601)	0.0018	99.6

10	*k*_*c*2_	(0.00028691, 0.46452)	0.074	99.6
11	*k*_*c*3_	(0.0029809, 1.07)	0.37	99.2
12	*k*_*degpin*_	(0, 0.736)	0.1	100.0

13	*k*_*a*1_	(0.10325, 1.629)	0.5	79.4
14	*k*_*d*1_	(0.0002, 0.01128)	0.0005	60.0
15	*k*_*degf*_	(0.0004, 0.0014042)	0.0005	10.0

16	*k*_*degc*_	(0, 0.00019312)	0.000022	100.0
17	*k*_*i*1_	(0.00155, 0.047406)	0.0026	40.4
18	$k_{e1f}$	(0, 0.00021787)	52E−6	100.0

19	$k_{e1c}$	(4.48E−05, 0.38702)	0.01	99.6
20	*k*_*i*2_	(6.84E−05, 0.0015916)	0.00067	89.8
21	*k*_*e*2_	(0, 0.016038)	3.35E−4	100.0

22	*h*	(0.68071, 2.4335)	2	21.7
23	*k*	(0.055, 0.11369)	0.065	15.4
24	*k*_*itria*_	(7.06E−08, 1.68E−07)	1.4E−7	19.8

25	*k*_*tria*_	(0.20438, 0.6)	0.5	20.0
26	*k*_*degtia*_	(4.98E−06, 0.00059088)	0.0003	97.0
27	*k*_*itra*_	(5.13E−08, 2.63E−06)	1.4E−7	63.4

28	*k*_*tra*_	(0.18323, 11.436)	0.5	63.4
29	*k*_*degta*_	(3.30E−05, 0.0020154)	0.00048	93.1

**Table 2:** The parameters with higher sensitivity scores, arranged in order of decreasing sensitivity.

Parameter		Description	Score
Num.	Name		
24	*k*	Nuclear NF-κB concentration at half-maximal transcription	1.17
4	*k*_*v*_	Ratio of cytoplasmic to nuclear volume	−1.01
23	*h*	Order of Hill function controlling transcription	−0.987
25	*k*_*itria*_	Inducible IκBα mRNA synthesis	−0.984
26	$\left(k_{\mathrm{tria}}\right)$	IκBα Translation rate	−0.984
2	totIKK	total IKK	0.914
7	*k*_*i*_	Spontaneous IKK inactivation	−0.912
27	*k*_*degtia*_	IkBa mRNA degradation	0.91
5	*k*_*p*_	IKKn production rate	0.87
8	*k*_*da*_	Constitutive A20 degradation	0.87
28	*k*_*itra*_	Inducible A20 mRNA synthesis	−0.86
29	*k*_*tra*_	A20 mRNA translation rate	−0.86
9	$k_{\mathrm{bA}20}$	A20 inactivation of IKK (All TNF conditions)	0.85
11	$k_{c_{3}}$	Catalysis of IKK.IkBa.NFkB trimer	0.79

## Appendix A Differential equations

The model studied in this paper is specified by the following ODEs:

$$\mathrm{NF}\prime =k_{d1}\cdot (\mathrm{totNFkB}-(\mathrm{nNF}+\mathrm{nIkBNF})/k_{v}-\mathrm{pIkBNF}-\mathrm{NF})-k_{a1}\cdot \mathrm{IkB}\cdot \mathrm{NF}-k_{i1}\cdot \mathrm{NF}+k_{\mathrm{degc}}\cdot (\mathrm{totNFkB}-(\mathrm{nNF}+\mathrm{nIkBNF})/k_{v}-\mathrm{pIkBNF}-\mathrm{NF})+k_{e1f}\cdot \mathrm{nNF}+k_{\mathrm{degpin}}\cdot \mathrm{pIkBNF}$$

$$\mathrm{IkB}\prime =k_{d1}\cdot (\mathrm{totNFkB}-(\mathrm{nNF}+\mathrm{nIkBNF})/k_{v}-\mathrm{pIkBNF}-\mathrm{NF})-k_{a1}\cdot \mathrm{IkB}\cdot \mathrm{NF}-k_{i2}\cdot \mathrm{IkB}+k_{e2}\cdot \mathrm{nIkB}-k_{\mathrm{degf}}\cdot \mathrm{IkB}+k_{\mathrm{tria}}\cdot \mathrm{tIkB}-k_{c2}\cdot \mathrm{IKK}\cdot \mathrm{IkB}$$

$$\mathrm{nNF}\prime =k_{d1}\cdot \mathrm{nIkBNF}-k_{a1}\cdot \mathrm{nIkB}\cdot \mathrm{nNF}+k_{v}\cdot k_{i1}\cdot \mathrm{NF}-k_{v}\cdot k_{e1f}\cdot \mathrm{nNF}$$

$$\mathrm{nIkB}\prime =k_{d1}\cdot \mathrm{nIkBNF}-k_{a1}\cdot \mathrm{nIkB}\cdot \mathrm{nNF}+k_{v}\cdot k_{i2}\cdot \mathrm{IkB}-k_{v}\cdot k_{e2}\cdot \mathrm{nIkB}-k_{\mathrm{degf}}\cdot \mathrm{nIkB}$$

$$\mathrm{nIkBNF}\prime =k_{a1}\cdot \mathrm{nIkB}\cdot \mathrm{nNF}-k_{d1}\cdot \mathrm{nIkBNF}-k_{v}\cdot k_{e1c}\cdot \mathrm{nIkBNF}$$

$$\mathrm{tIkB}\prime =k_{\mathrm{itria}}\cdot \frac{{\mathrm{nNF}}^{h}}{{\mathrm{nNF}}^{h}+k^{h}}-k_{\mathrm{degtia}}\cdot \mathrm{tIkB}$$

$$\mathrm{IKKn}\prime =k_{p}\cdot (\mathrm{totIKK}-\mathrm{IKK}-\mathrm{IKKn})\cdot \frac{k_{\mathrm{bA}20}}{k_{\mathrm{bA}20}+A20\cdot T_{R}}-T_{R}\cdot k_{a}\cdot \mathrm{IKKn}$$

$$\mathrm{IKK}\prime =T_{R}\cdot k_{a}\cdot \mathrm{IKKn}-k_{i}\cdot \mathrm{IKK}$$

$$\mathrm{tA}20\prime =k_{\mathrm{itra}}\cdot \frac{{\mathrm{nNF}}^{h}}{{\mathrm{nNF}}^{h}+k^{h}}-k_{\mathrm{degta}}\cdot \mathrm{tA}20$$

$$A20\prime =k_{\mathrm{tra}}\cdot \mathrm{tA}20-k_{\mathrm{da}}\cdot A20$$

$$\mathrm{pIkBNF}\prime =k_{c3}\cdot \mathrm{IKK}\cdot (\mathrm{totNFkB}-(\mathrm{nNF}+\mathrm{nIkBNF})/k_{v}-\mathrm{pIkBNF}-\mathrm{NF})-k_{\mathrm{degpin}}\cdot \mathrm{pIkBNF}$$

## Appendix B The low-dose oscillatory regime is observable

The low-dose oscillatory regime is shown in Fig. B1.

## Appendix C Two-parameter bifurcation diagrams

This section includes the remaining two-parameter bifurcation diagrams for

$$\left(T_{R},p)\in [0,1]\times [0,2p^{⋆}\right]$$

where *p* is one of the model's parameters and $p^{⋆}$ refers to the value used by Ashall et al. (2009). Each point on the curves is a Hopf bifurcation (HB) point and the curves divide the parameter space into two types of regions: limit cycle (marked by 1) and steady state (marked by 2) Figs. C1–C6.
